# A Case of Sinonasal Undifferentiated Carcinoma with Brain Metastases

**DOI:** 10.7759/cureus.2320

**Published:** 2018-03-13

**Authors:** Julianna Sienna, Nhu-Tram Nguyen, Julie Arsenault, Ian Hodson, Brandon Meyers

**Affiliations:** 1 Resident - Radiation Oncology, Juravinski Cancer Centre-McMaster University; 2 Division of Radiation Oncology, McMaster University-Juravinski Cancer Centre, Hamilton, ON; 3 Department of Oncology, Dr. Georges-L.-Dumont University Hospital Centre; 4 Department of Radiation Oncology, Juravinski Cancer Centre-McMaster University; 5 Department of Medical Oncology, Juravinski Cancer Centre-McMaster University

**Keywords:** sinonasal undifferentiated carcinoma, radiosurgery, hyperfractionated radiotherapy, split course radiotherapy, palliative radiotherapy, induction chemotherapy, stereotactic radiosurgery, snuc

## Abstract

Sinonasal undifferentiated carcinoma (SNUC) is a rare aggressive neoplasm arising in the nasal cavity and paranasal sinuses. We report a unique case of an 80-year-old man who presented with a locally advanced SNUC involving the ethmoid, sphenoid, and maxillary sinuses and bilateral lymph nodes, clinical T4N2M0. Given his age and the initial extent of his primary tumour, he was treated with neoadjuvant chemotherapy followed by chemoradiation with a split course of 50 Gray (Gy) in 40 fractions delivered twice a day. Four months after his treatments, he developed a recurrence at the left lower eyelid and left frontal sinus, intrabdominal metastases, and a left cerebellar metastasis. A single fraction of 22 Gy was delivered to the cerebellar lesion using stereotactic radiosurgery. He survived 17 months from the initial presentation. We review the available literature regarding treatment of brain metastases and use of hyperfractionated radiotherapy in this rare head and neck cancer.

## Introduction

Sinonasal undifferentiated carcinoma (SNUC) is a rare and aggressive form of head and neck cancer that arises from the nasal cavity and paranasal sinuses. Studies of this cancer have been limited to case reports and series, retrospective reviews, and two small meta-analyses [1-2]. As a result, reproducible evidence and reliable guidelines for optimal treatment of SNUC have not been established. This has been further confounded by heterogeneity in the severity of disease at presentation and availability of surgical and radiation treatment techniques both geographically and temporally.

Despite treatment that may include surgery, radiation, and/or chemotherapy, long-term outcomes are quite poor. Median disease-free survival is estimated at 12.7 months for patients at all stages and treatment modalities; the five-year survival is estimated to be about 6% [2]. Recent literature reviews have provided conflicting views on optimal treatment for SNUC, with some suggesting a trimodality approach, while others question the benefit of surgical resection in these aggressive cancers. A meta-analysis demonstrated a survival benefit when combining surgery with radiotherapy and/or chemotherapy compared to surgery alone, and this difference was significant in patients with Kadish Stage C disease [1]. However, there was no statistically significant improvement in survival with trimodality treatment (surgery + radiation + chemotherapy) compared to surgery, plus chemotherapy, or surgery, plus radiotherapy, alone. Intracranial extension, orbital, or dural involvement are no longer considered contraindications to surgical management, with many patients receiving preoperative concurrent chemoradiation with or without induction chemotherapy [3-4]. Patients with unresectable disease have been treated with definitive chemoradiation or induction chemotherapy followed by concurrent chemoradiotherapy [5]. 

We report on a case of SNUC that presented as locally advanced disease and discuss our initial management, as well as management of distant recurrence. Specifically, we comment on the use of hyperfractionated radiation treatment in the initial management and our use of stereotactic radiosurgery in treating brain metastases.

## Case presentation

Presentation

In August 2013, a previously healthy 80-year-old gentleman with a remote 30 pack-year smoking history presented with a mass arising from the left anterior nasal cavity. At presentation, there was also a mass in the nasopharynx that was felt to be contiguous with the nasal cavity tumour and extensive palpable bilateral cervical lymphadenopathy.

Investigations

**Figure 1:**
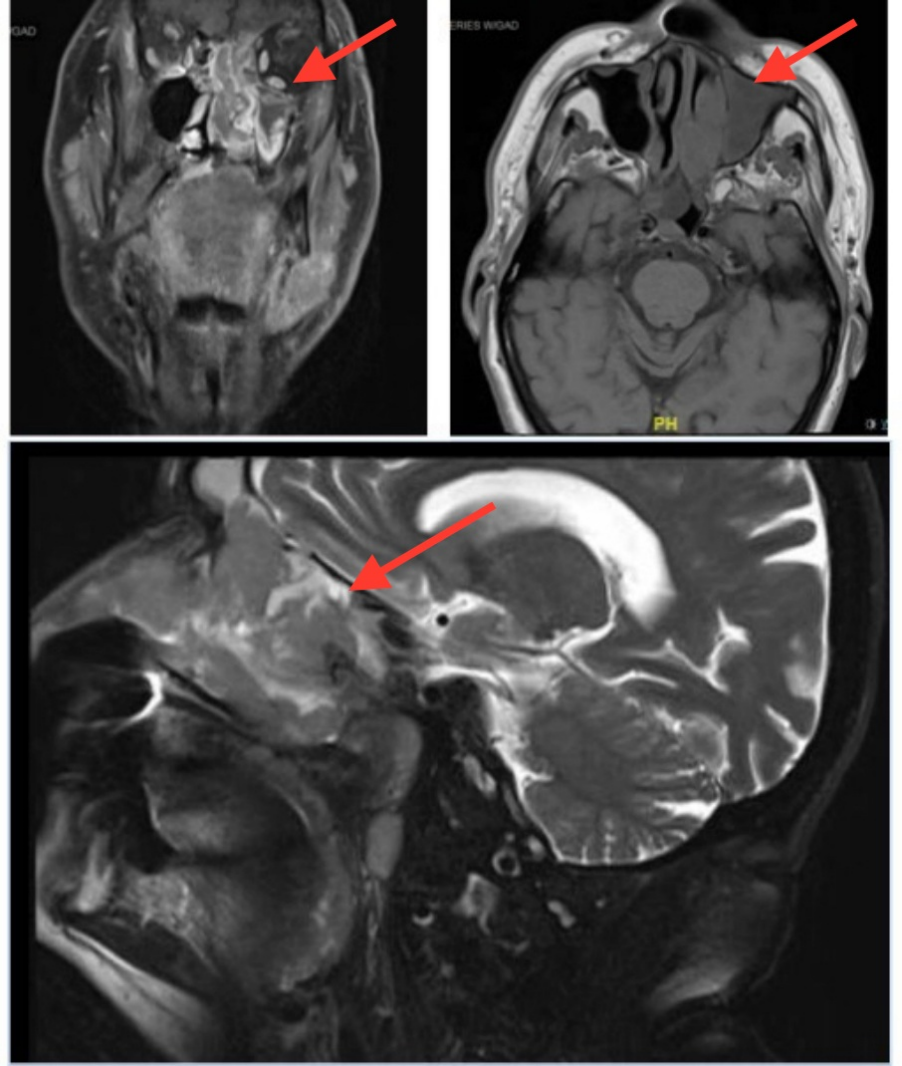
Pre-treatment magnetic resonance imaging (MRI) Clockwise from top left: coronal T1 with gadolinium, axial T1, and sagittal T2

Staging computed tomography (CT) and magnetic resonance imaging (MRI) demonstrated a 4.5 centimetre (cm) x 7 cm x 2.7 cm mass centered in the left naris and nasopharynx with involvement of the left frontal, ethmoid, and bilateral sphenoid and maxillary sinuses extending into the base of the skull with erosion of the cribriform plate and fovea ethmoidalis (Figure 1). There were enlarged lymph nodes in the right neck levels one to three, and left neck levels one to five. There was no involvement of adjacent meninges or brain parenchyma.

**Figure 2:**
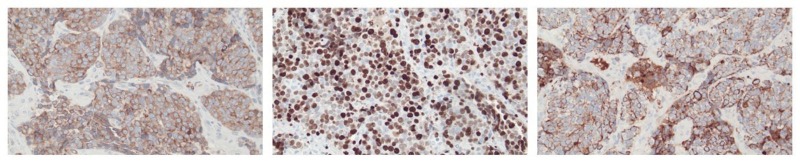
Histologic stains From left to right: B-cell lymphoma antigen 2 (BCL-2), Ki-67, and cytokeratin 7 (CK7)

A biopsy of the nasal cavity mass showed a high-grade malignant neoplasm, favoured to represent a sinonasal undifferentiated carcinoma, with immuno-histochemistry demonstrating a strong positivity for cytokeratin 7 (CK7), and B-cell lymphoma antigen 2 (BCL-2). Ki-67 was 80-90% (Figure 2). A pathology review confirmed sinonasal undifferentiated carcinoma.

Treatment

Due to the extent of his disease, the patient initially received three cycles of induction cisplatin, 75 milligram (mg)/metre (m)^2^ at three-week intervals. An interval MRI at two months showed a significant decrease in the nasopharyngeal mass and the lymphadenopathy. He subsequently received concurrent chemoradiation with split-course radiation therapy. The radiotherapy consisted of two courses of 25 Gray (Gy) in 20 fractions delivered twice daily over 10 days, with a four-week rest in between. Single-agent cisplatin (75 mg/m^2^) chemotherapy was delivered on the first day of each course of radiation. Disease status and treatment toxicity were assessed following the first half of the split course radiotherapy, and the patient successfully completed the intended treatment. The patient did not experience any Grade 3 or 4 acute toxicity. He experienced Grade 2 fatigue, weight loss, and dysphagia on the Common Terminology Criteria for Adverse Events (CTCAE), version 4.

Outcome and follow-up

The patient completed chemoradiation in February 2014. Two months later, the restaging MRI showed near complete response of the primary disease, with minimal residual disease in the maxillary and sphenoid sinuses and no residual lymphadenopathy.

He recurred in August 2014 at the left lower eyelid. A re-staging CT showed findings highly suggestive of peritoneal lymphadenopathy and possible adrenal metastases. An MRI in September 2014 showed a left cerebellar lesion and confirmed the left lower eyelid and left frontal sinus masses. A biopsy of the left lower eyelid demonstrated a poorly differentiated malignant neoplasm favouring metastatic undifferentiated sinonasal carcinoma. An immunohistochemical profile was similar to the initial tumour: strongly positive for CK7, CK AE1/AE3, and BCL-2, and negative for CK5/6, p63, and CK20. Ki67 showed intense staining highlighting approximately 90% of cells.

Given the patient's age and the short interval to disease recurrence, it was felt he should be treated with a second line, single agent weekly paclitaxel (80 mg/m^2^ on days 1, 8, and 15 of a 28-day cycle). Clinically, there appeared to be a response to the eyelid lesion (1 cm x 3 cm from 3 cm x 3 cm). He also underwent stereotactic radiosurgery (SRS) to the cerebellar metastasis to a dose of 2,200 cGy in a single fraction. Unfortunately, he had to stop chemotherapy after one and a half cycles due to a decline in his performance status. In the following three weeks, the lesion on the eyelid grew back rapidly, and he developed fulminant liver failure secondary to hepatic metastases leading to hospital admission. He died two weeks later.

## Discussion

Our patient, who presented with locally advanced disease, was treated with induction chemotherapy and concurrent chemoradiation, with radiation delivered using a hyper-fractionated split course. Despite a near complete response following his initial treatments, the disease rapidly recurred both locally and distantly (within six months of completing chemoradiation). He developed a cerebellar metastasis that was treated with stereotactic radiosurgery. In total, he survived 17 months from initial presentation.

Brain metastases

Brain metastases are a rare outcome, estimated to occur in 5% of cases in an already very rare cancer [2]. One of the difficulties in addressing this condition involves the propensity for SNUC to invade the brain, either at presentation or when it recurs. We conducted a systematic review of all published literature on SNUC using OVID search criteria "sinonasal undifferentiated carcinoma" or “sinonasal undifferentiated” or "SNUC" before August 2016. In total, 200 abstracts were obtained, and applicable studies were then reviewed in full. In our review of the literature, we found references in eight publications to metastases to the brain or central nervous system. Of the eight studies identified, treatment of brain metastases was detailed for two patients [3, 6]. One of these patients received no treatment and died of disease two months after presentation. The other patient was treated with surgery and SRS and died of disease 48 months after presentation. An additional two patients with brain involvement were described by Jeng, but it is not clear whether they had direct brain invasion or metastases [7].

Hyperfractionated radiotherapy

In this case, a hyperfractionated method was chosen in an attempt to reduce what was felt would likely be excessive toxicity, given the extensive volume. The decision to split the course was a pragmatic one. While a cure was felt to be possible, there remained a significant concern for how the patient would tolerate treatment, given his age. The split course method allowed for assessment of tolerance before starting the second half.

A meta-analysis of altered fractionated radiotherapy (RT) in squamous cell carcinoma of the head and neck showed improved overall survival with altered fractionation compared to standard fractionation, with the greatest benefit seen with hyperfractionation; however, there was no break in between [8]. In reviewing all cases of SNUC in the literature, we identified 13 cases of SNUC tumours treated with hyperfractionated RT. Two of the 13 patients did not undergo surgery, and of those, one was treated with concurrent chemotherapy. That patient died with the disease at 18 months. RT doses ranged from 59.4 - 60 Gy (neoadjuvant), 70.8 - 74.8 Gy (definitive), and 50 - 74.4 Gy (adjuvant). Survival data from these cases are summarized in Table 1. 

**Table 1:** Treatment and outcomes of cases of SNUC treated with hyperfractionated RT N: number; SNUC: sinonasal undifferentiated carcinoma; HFRT: hyperfractionated radiotherapy; RT: radiotherapy; CCRT: combined chemo-radiotherapy; Gy: Gray; BID: twice daily treatment; 5FU: 5-fluorouracile; DM: distant metastasis; mos: months; LR:  local recurrence; RR: regional recurrence; Pt: patient; AWD: alive with disease; DWD: died with disease; NED: no evidence of disease

Author (Year)	Pitman et al. (1995) [4]	Esposito et al. (2006) [9]	Tanzler et al. (2008) [10]		
N in series	1	4	15		
N with SNUC	1	1	15		
N with SNUC + HFRT	1	1	11		
Treatment	Neoadjuvant RT $\rightarrow$ 4 cycles chemo (etoposide and cisplatin) $\rightarrow$ surgery	Surgery $\rightarrow$ adjuvant CCRT	Definitive RT (n = 2)	Neoadjuvant CCRT $\rightarrow$ surgery (n = 2)	Surgery $\rightarrow$ adjuvant RT, all node negative (n = 7)
Indication	Neoadjuvant	Adjuvant	Definitive	Neoadjuvant	Adjuvant
Dose	64 Gy	50 - 60 Gy	70.8 - 74.8 Gy	59.4 - 60 Gy	62.4 - 74.4 Gy
Fractions	40	--	--	--	--
Frequency	BID	BID	BID	BID	BID
Concurrent Chemo	No	Yes	Yes (n = 1)	Yes	Yes (n = 2)
CCRT regimen	--	5-FU and cisplatin	Carboplatin and taxol	Cisplatin +/- taxol	Cisplatin
Outcomes	DM (liver) at 7 mos (no LR or RR). Pt opted for palliative chemo $\rightarrow$ moderate response. No survival data	Reoperation 15 mos after first surgery. AWD at 24 mos	1 = DWD at 18 mos (LR + RR at 16 mos); 1 = NED at 84 mos (dural recurrences at 10 and 16 mos)	1 = AWD at 22 mos (DM at 9 mos); 1 = DWD at 38 mos (RR at 12 mos, DM at 21 mos)	4 = NED at 12, 14, 19, and 128 mos; 1 = DWD at 11 mos, 2 died of other causes (20, 47 mos)

## Conclusions

The optimal treatment of SNUC remains unclear. Despite multimodality treatment, outcomes of patients with SNUC remain generally poor with patients rarely surviving beyond two years. Our experience would suggest that in patients with bulky disease for whom a conventionally fractionated course of chemoradiation over seven weeks would be challenging, a split-course of chemoradiation could be considered with or without induction chemotherapy.

In our patient, the use of induction chemotherapy was key as initial volumes were extensive and it allowed us to assess response in this aggressive disease. This approach was associated with an excellent tumour response, reasonable survival (similar to other published studies), and was well tolerated because of the four week break period. Split-course radiotherapy would also allow the opportunity to consider adaptive radiation to decrease doses to organs at risk in areas not clinically involved at the time of diagnosis. It is unclear whether this gentleman would have benefited from surgical resection of his remaining disease once his disease was stable, and whether this may have provided even more durable response to treatment.

We recommend the creation of a rare head and neck disease database in order to allow for more standardized data collection and thus hopefully a tool to better evaluate outcomes in this disease.
